# Mid-luteal estradiol levels of poor/good responders and intracytoplasmic sperm injection

**DOI:** 10.12669/pjms.331.11692

**Published:** 2017

**Authors:** Rehana Rehman, Sundus Tariq, Saba Tariq, Faisal Hashmi, Mukhtiar Baig

**Affiliations:** 1 Dr. Rehana Rehman, MBBS, M.Phil, PhD Physiology, Aga Khan University, Karachi, Pakistan; 2 Dr. Sundus Tariq, MBBS, M.Phil. Assistant Professor of Physiology, University Medical & Dental College, Faisalabad, Pakistan; 3 Dr. Saba Tariq, MBBS, M.Phil. Assistant Professor of Pharmacology & Therapeutics University Medical & Dental College, Faisalabad, Pakistan; 4 Dr. Muhammad Faisal Hashmi, MBBS. Postgraduate Trainee, Services Hospital Lahore, Pakistan; 5 Dr. Mukhtiar Baig, MBBS, M.Phil, PhD. Professor of Clinical Biochemistry, Faculty of Medicine, Rabigh, King Abdulaziz University, Jeddah, Saudi Arabia

**Keywords:** Good responders, ICSI, Infertility, Mid-luteal E2, Poor responders

## Abstract

**Objective:**

To assess mid-luteal estradiol (E2) levels in poor and good responders and determine its effect on the outcome after intracytoplasmic sperm injection (ICSI).

**Methods:**

The current study was carried out in females who underwent ICSI from June 2011 to September 2013 in “Islamabad Clinic Serving Infertile Couples”. They were categorized into good and poor responders on the basis of female age ≤40 years, basal follicle stimulating hormone ≤12 mIU/ml, and antral follicle count >5, respectively. Their mid-luteal E2 measured on the day of embryo transfer was stratified into groups (A-E) on the basis of 20th, 40th, 60th and 80th percentile values. The outcome was categorized into non-pregnant with beta human chorionic Gonadotrophin (hCG) 5-25 m IU/ml, and clinical pregnancy with beta hCG>25 m IU/ml.

**Results:**

The conception rate was 12% (63/513) in poor responders and 72% (237/329) in good responders respectively. The mid-luteal E2 levels were higher in conception as compared to non-conception cycles (p<0.001) in good and poor responders.

**Conclusion:**

Maximum pregnancies in poor and good responders (53% and 98% respectively) with mid-luteal E2 levels above 80^th^ percentiles confirm the role of the increase in mid-luteal E2 for augmentation in conception rate of females after ICSI.

## INTRODUCTION

Infertility refers to the failure to conception by the couple and is perceived as a multifactorial syndrome in all cultures and societies.^1^ Out of the offered treatment procedures in reproductive clinics, in vitro fertilization (IVF) and intracytoplasmic sperm injection (ICSI) are the advanced reproductive techniques (ART). In these procedures, ovaries are down-regulated and then stimulated to produce eggs, which retrieved, and then microinjected with spermatozoa.^2^,^3^ The success of the procedure depends on quality of embryos and endometrial receptivity offered at the time of implantation.^4^

One of the elementary steps for success depends on a number of eggs obtained at the end of controlled ovarian stimulation (COS). Females show a different response to stimulation on the basis of which the terms, “poor responders,” and good responders are described on varying criteria. The term “poor 888 responders”, is use for females, when limited numbers of eggs retrieved after COS^5^ resulting in lesser number of embryos to select and with reduced pregnancy and live birth rates and higher chances of miscarriage. According to European Society for Human Reproduction and Embryology (ESHRE), poor ovarian response (POR) is designated with at least two of the following three features; “advanced maternal age or any other risk factor for POR, a previous POR with maturation of 3 oocytes in the previous cycle by COS protocol an abnormalovarian reserve test i.e. “antral follicle count (AFC) less than 5–7 follicles or anti-Mullerian hormone (AMH) below 0.5–1.1 ng/ml”.^6^ Discovery and trials of new hormonal preparations have resulted in number of modifications and variations in stimulation protocols with little improvement in oocyte and embryo quality and hence pregnancy outcome in poor responders.

The estradiol (E2) produced by the granulosa cells of the ovaries upregulates progesterone receptors for preparation of blastocysts implantation in normal and assisted conceptions.^7^ Peak E2 is estimation of hormoneon the day of human chorionic Gonadotrophin (hCG) that gives an indirect evidence of ovarian responsiveness and gives an insight to success of treatment procedures.^3^,^4^ Oestrogen and progesterone receptors both increase endometrial glands and stroma during follicular and early luteal phases of a normal menstrual cycle. Luteal phase E2 stimulates progesterone receptors and also proliferate endometrial gland resulting in hypertrophy and hyperplasia of endometrial epithelia which may or may not favor implantation.^8^

Pituitary down-regulation for COS reduces E2as well as P levels in the luteal phase.^4^ This decline of serum E2 and progesterone levels in mid-luteal phase of normal menstrual cycle can adversely affect the results of implantation and successful pregnancy during IVF/ICSI cycles and increase rate of non-conception and biochemical pregnancies.^9^-^11^

In spite of a variety of protocols and supportive therapies, poor responders continue to represent a challenge to IVF experts.^12^ The addition of E2 in the luteal phase for improvement in the rate of pregnancy or implantation rates in the selected cases of poor responders is still a subject of debate.^13^ In this study, we wanted to assess mid-luteal estradiol (E2) levels in poor and good responders and compare its effect on the outcome of ICSI in our local population. The results of the study may help in identification of the patients (poor/good responders) who could benefit with the supplementation of E2 supplements.

## METHODS

The current study was conducted from June 2011 to September 2013 after ethical approval from Institutional Review Board of “Islamabad Clinic Serving Infertile Couples” All clinical investigations followed principles of Declaration of Helsinki. Females included in the study had primary infertility for more than two years with the normal menstrual cycle of 25 ± 7days. The Baseline investigations included; (day three follicle stimulating hormone;FSH) and antral follicle count (AFC) done by the transvaginal scan. All females with polycystic ovaries and anatomical or morphological abnormalitieswere excluded.

These patients had down-regulation with long protocol by use of injection decapeptide, (Ferring, Copenhagen NV) and COS with recombinant FSH for a period of 12± two days. The ovulation induction (OI) was done by injection of 10,000 IU of human chorionic gonadotropin (HCG) after confirmation of maturity of at least two follicles acquiring a size of 18 -20 mm, The venous sample was obtained for measurement of peak estradiol E2 on OI. Oocyte retrieval was performed on 35-hour after OI by the transvaginal route under ultrasound guidance followed by embryo transfer three days after the procedure. Mid-luteal estimation of E2 was done on the day of transfer and groups (A-E) were stratified based on percentile values 20th, 40th, 60th and 80th percentile with 841, 948, 997and ≥1081.2 pg/ml E2 levels. Clinical pregnancy was demarcated by the existence of a gestational sac with cardiac activity observed by TVS at the 7th week of gestation.

### Statistical Analysis

The data was analyzed on SPSS 21, and continuous variables were represented by mean and standard deviation. Student’s t test was employed for comparing two groups (poor/good responders) and p<0.0.5 considered significant.

## RESULTS

Females enrolled in the study (842) were categorized into groups of poor (513) and good (329) respectively with an age range of 32.25±4.3 years. Conception occurred in 365 patients, 100 (27%) poor and 265(73%) good responders, respectively. The comparison of characteristics in overall, poor and good responders are given in Table-I. The difference in several variables between poor and good responders, like age of menarche, body mass index (BMI), antral follicle count, estradiol before treatment, the length of stimulation, preovulatory follicle count, the number of fertilized oocyte cleaved and transferred embryos and endometrial thickness was significant. Table-II shows peak and mid luteal E2 in all patients, good responders, and poor responders. The peak E2, mid luteal E2 were significantly high in pregnant females in all three groups (p<0.05), while peak/mid-luteal E2 ratio was significantly lower in all three groups (p<0.05).

**Table-I:** Comparison of characteristics in overall, poor and good responders.

Variables	Overall (842)	Poor responders (513)	Good responders (329)	P value

	Mean ±SD	Mean ±SD	Mean ±SD	
Duration of infertility	7.11 ± 3.9	6.97 ± 3.6	7.3 ± 4.2	0.22
Female age	32.11 ± 4.6	31.97 ± 4.6	32.3 ± 4.7	0.31
Age of menarche	14.05±1.17	14.188±1.2	13.877±1.0	0.000
Estradiol before treatment(pg/ml)	214.74±145.6	190.367±130.6	246.709±157.9	0.000
Antral Follicle Count	14.66±2.80	14.938±2.3	14.295±2.5	0.001
BMI	24.24 ± 3.7	24.51 ± 3.7	23.9 ± 3.7	0.01
Length of stimulation	14.34 ± 1.0	14.44 ± 1.0	14.2 ± 1.0	<0.001
PFC	7.8 ± 1.9	7.23 ± 2.0	8.56 ± 1.4	<0.001
No. of oocytes/patient	7.69 ± 1.7	7.11 ± 1.7	8.45 ± 1.2	<0.001
No. of oocytes Metaphase II	7.13 ± 2.0	6.2 ± 2.0	8.34 ± 1.1	<0.001
No. of oocytes fertilized	5.95 ± 1.6	5.18 ± 1.6	6.96 ± 0.7	<0.001
Endo. Lining	8.6 ± 3.4	7.81 ± 3.3	9.63 ± 3.3	<0.001
No. of transferred embryos	1.62 ± 0.6	1.58 ± 0.6	1.68 ± 0.6	0.01

BMI=Body mass index, PFC= Preovulatory follicle count, Endo= endometrial thickness in mm, No.= number.

**Table-II:** Peak and mid luteal E2 in all patients, good responders and poor responders.

Variables	Pregnant	Not pregnant	P value
***Peak and mid luteal E2 in all patients***			
Peak E2 (pg/ml)	2556.51 ± 173.1	2404.27± 157.6	<0.001
Mid luteal E2 (pg/ml)	1121.94 ±139.9	876.61 ± 98.8	<0.001
Peak/mid luteal	2.30 ± 0.3	2.77 ± 0.3	<0.001
***Peak and mid luteal E2 in good responders***			
Peak E2 (pg/ml)	2556.507 ± 173.13	2404.272 ± 157.5	<0.001
Mid luteal E2 (pg/ml)	1121.943 ± 139.87	876.61 ± 98.8	<0.001
Peak/mid luteal E2	2.305 ± 0.26	2.77 ± 0.3	<0.001
***Peak and mid luteal E2 in poor responders***			
Peak E2 (pg/ml)	2440.99 ± 227.2	2143.01 ± 286.4	<0.001
Mid luteal E2 (pg/ml)	1069.42 ±114.0	907.06 ± 128.0	<0.001
Peak/mid luteal E2	2.29 ± 0.2	2.40 ± 0.4	0.04

E2= estradiol, Values are Mean ± SD.

**Fig. 1:**
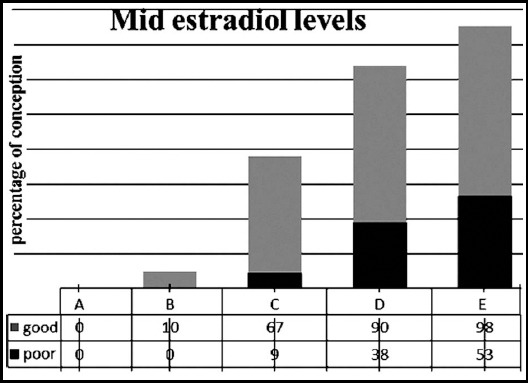
Comparison of pregnancy rates based on mid-lutealestradiol levels in poor and good responders.

Fig.1 shows the comparison of clinical pregnancy rate in poor and good responders on the basis of mid E2 levels. Based on stratification in groups (A-E), Group A comprised of 123 poor and 45 good responders, all failed to conceive. In-group B, 10/60(17%) good responders conceived out of 168 females. The pregnancy rate was 9.5%(12/126), 66.7%(30/45) in poor and good responders of Group C. Females (164) with mid-luteal levels ≥ 997 pg/ml in Group D had 38%(30/78) and 90%(78/86) pregnancy rate in poor and good responders respectively. In-group comprising of 171 females 123/126 good responders (98%) conceived in comparison to 21/45, 53% poor responders. The conception rate in females with mid-luteal E2 levels below 20^th^ percentile was zero in both poor and good responders, but this rate gradually increased in with mid-luteal E2 levels above 40^th^, 60^th^ and 80^th^ percentiles, with maximum conception rate above 80^th^ percentile indicating that high mid-luteal E2 levels indicate outcome of conception in ICSI especially in good responders.

Mid-luteal E2 groups stratified on the basis of percentile values; 20^th^, 40^th^, 60^th^ and 80^th^ percentile with 841, 948, 997and 1081.2 pg/ml.

## DISCUSSION

The ART clinics try their level best to plan treatment plans with minimum complicationsin terms of selection of patients, a number of visits, type of protocol, injections for stimulation; their cost vs. side effects in comparison to patient’s satisfaction and clinical pregnancy rate. The measurement of peak and luteal E2 is done in these patients keeping in mind its importance for proliferation of endometrium and up-regulation of progesterone receptors required in IVF and ICSI cycles.^14^

Studies have shown that high peak and mid luteal E2 can predict the success of treatment after ICSI by the provision of optimal environment required for implantation of fertilized ovum and accomplishment of clinical pregnancy.^7^,^8^ In our study, peak and mid luteal E2 levels were higher in pregnant females as compared to the non-pregnant group which is similar to other studies.^15^

Similarly, trends were seen in studies showing significantly high E2 and progesterone in females who have conceived and with on-going pregnancy as compared to non-conception group and females with miscarriages respectively, showing that both E2 and progesterone in mid-luteal phase can predict clinical pregnancy outcome in IVF/ICSI cycles.^16^

On the contrary, studies have established no role of mid-luteal E2 in the improvement of pregnancy.^13^-^15^,^17^,^18^ There are also contradictory randomized controlled trials, in which addition of E2 through oral medications in luteal phase did not improve IVF/ICSI outcomes.^19^

In our study, peak and mid luteal E2 levels were significantly high in pregnant as compared to non-pregnant females in both poor and good responders, while peak to mid luteal estradiol ratio was low in pregnant as compared to non-pregnant females in both poor and good responders. Studies have also shown significantly high mid-luteal estradiol levels in pregnant females in good responders but no such significance was seen in poor responders.^4^

The conception rate in females with mid-lutealE2 levels below 20^th^ percentile was zero in both poor and good responders, but this rate gradually increased in good responders with mid-luteal E2 levels above 40^th^, 60^th^, and 80^th^ percentiles, with maximum conception rate above 80^th^ percentiles indicating that high mid-luteal E2 levels indicate outcome of conception in ICSI especially in good responders.

## CONCLUSION

Maximum pregnancies in poor and good responders (53% and 98% respectively) occurred with mid-luteal E2 levels above 80^th^ percentiles. The results confirm the role of the increase in mid-luteal E2 for augmentation in conception rate of females after ICSI. Further experimental trials are required to explore the usefulness of E2 supplementation for support of conception after ICSI.
